# A metabolic link to skeletal muscle wasting and regeneration

**DOI:** 10.3389/fphys.2014.00032

**Published:** 2014-02-03

**Authors:** René Koopman, C. Hai Ly, James G. Ryall

**Affiliations:** ^1^Clinical Nutrition and Muscle and Exercise Metabolism Group, The University of MelbourneMelbourne, VIC, Australia; ^2^Stem Cell Metabolism and Regenerative Medicine Group, Basic and Clinical Myology Laboratory, Department of Physiology, The University of MelbourneMelbourne, VIC, Australia

**Keywords:** metabolism, satellite cells, stem cells, cell fate, glycolysis

## Abstract

Due to its essential role in movement, insulating the internal organs, generating heat to maintain core body temperature, and acting as a major energy storage depot, any impairment to skeletal muscle structure and function may lead to an increase in both morbidity and mortality. In the context of skeletal muscle, altered metabolism is directly associated with numerous pathologies and disorders, including diabetes, and obesity, while many skeletal muscle pathologies have secondary changes in metabolism, including cancer cachexia, sarcopenia and the muscular dystrophies. Furthermore, the importance of cellular metabolism in the regulation of skeletal muscle stem cells is beginning to receive significant attention. Thus, it is clear that skeletal muscle metabolism is intricately linked to the regulation of skeletal muscle mass and regeneration. The aim of this review is to discuss some of the recent findings linking a change in metabolism to changes in skeletal muscle mass, as well as describing some of the recent studies in developmental, cancer and stem-cell biology that have identified a role for cellular metabolism in the regulation of stem cell function, a process termed “metabolic reprogramming.”

## Introduction

Metabolism is loosely defined as the collection of enzymatic reactions essential for life, and can be catabolic/anabolic and exothermic/endothermic in nature. While in a constant state of flux, these reactions reach equilibrium (homeostasis) and are maintained in the absence of altered energy supply or demand. However, a sustained change in metabolism can have serious implications for an individual and can lead to an increase in both morbidity and mortality. In the context of skeletal muscle, altered metabolism is associated with numerous pathologies and disorders, including diabetes, obesity, Pompe's disease, McArdle disease and numerous mitochondrial disorders (Angelini and Semplicini, 2010; Raben et al., 2012; Russell et al., 2013), while many skeletal muscle pathologies have secondary changes in metabolism, including cancer cachexia, age-related muscle wasting and weakness (termed sarcopenia) and the muscular dystrophies (Ryall et al., 2008; Russell et al., 2013). Furthermore, the importance of cellular metabolism in the regulation of skeletal muscle stem cells is beginning to receive significant attention (Ryall, 2013). Thus, it is clear that skeletal muscle metabolism is intricately linked to the regulation of skeletal muscle mass and regeneration. The aim of this review is to discuss some of the recent findings regarding the role of metabolic dysfunction in skeletal muscle wasting and weakness, as well as to highlight potential novel therapeutic targets for future drug discovery and development. Finally, as cellular metabolism is beginning to receive increased attention in the regulation of stem cell identity, we discuss some of the implications for the regulation of skeletal muscle stem cell activity and regeneration following injury. However, before addressing these topics in detail, a brief overview of the major metabolic pathways will be discussed.

## Cellular metabolism in skeletal muscle

Energy in the form of adenosine triphosphate (ATP) is essential for cells to conduct the processes necessary for life, and depletion of ATP can lead to necrosis or apoptosis (Tsujimoto, 1997). The conversion of ATP to adenosine diphosphate (ADP) or adenosine monophosphate (AMP) and inorganic phosphate (P_i_) is exothermic and liberates energy that can be harnessed to fuel enzymatic reactions. Cellular ATP is derived from the breakdown of fats (via fatty acid oxidation, FAO), carbohydrates (via glycolysis) and proteins (via proteolysis) to pyruvate and/or acetyl CoA which, in the presence of oxygen, can be converted to ATP in the mitochondria via oxidative phosphorylation (OXPHOS). The majority of ATP is generated via glycolysis in the cytoplasm and OXPHOS in the mitochondria, with the relative contribution of each process dependent on a range of factors, including substrate and oxygen availability, and cellular energy demand (Salway, 2012).

Briefly, FAO involves an energy consuming reaction which converts FA and Co-enzyme A (Co-A) to fatty acyl-CoA (FA-CoA) and is catalyzed by the enzyme fatty acyl-CoA synthetase. FA-CoA cannot be directly transported across the mitochondrial inner membrane and must first be converted to an acyl carnitine derivative and then reconverted to FA-CoA inside the mitochondria. FAO involves a stepwise process of dehydrogenation of acyl-CoA to acetyl-CoA which can then be metabolized by the tricarboxylic acid cycle (TCA) and the mitochondrial electron transport chain (ETC, Salway, 2012, Figure 1).

**Figure 1:**
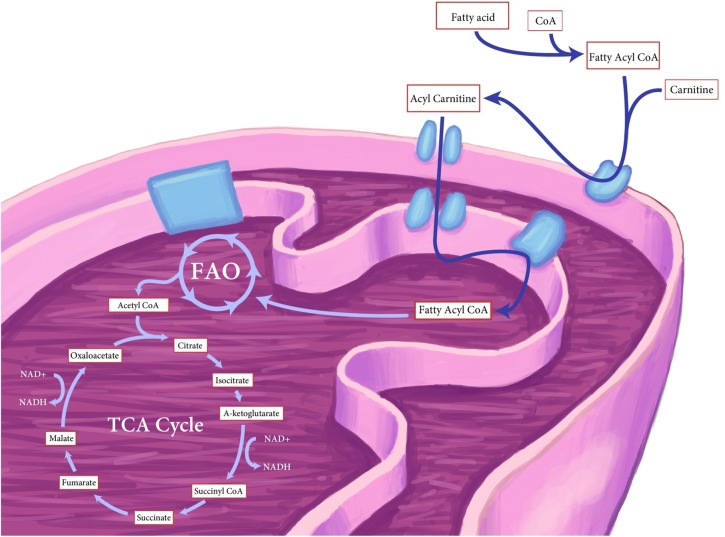
**Schematic of the process of fatty-acid oxidation (FAO) in cells**. Fatty-acid-CoA (FA-CoA) is converted to an acyl carnitine derivative in the mitochondrial membrane, acylcarnitine is then converted back to FA-CoA within the mitochondria where it undergoes a series of dehydrogenation reactions to form acetyl-CoA. Acetyl-CoA, in turn enters the tricarboxylic acid (TCA) cycle to generate NADH to drive complex I, and succinate to drive complex II of the mitochondrial electron transport chain.

Glucose is metabolized by almost all organisms in a cytosolic process termed glycolysis which yields two molecules of ATP per molecule of glucose. Circulating glucose enters a cell predominantly via a family of transmembrane glucose transporters (GLUT1-11), with several isoforms each being specific to certain tissues. Once inside the cell, glucose is converted to glucose-6-phosphate (G6P) by hexokinase in an ATP consuming reaction, following which G6P is converted first to fructose-6-phosphate (F6P) and then to fructose-1-6-bisphosphate (F1,6BP). The conversion to F1,6BP is irreversible and is considered the step at which glucose is committed to glycolysis (Lunt and Vander Heiden, 2011). This reaction is catalyzed by phosphofructokinase 1 (PFK1); an enzyme allosterically controlled by levels of ATP, such that abundant levels of ATP inhibits PFK1. Cleavage of F1,6BP generates two molecules of glyceraldehyde-3-phosphate (G3P), which are then converted to phosphoenolpyruvate (PEP). The final step of glycolysis involves the conversion of PEP to pyruvate to release ATP; a reaction catalyzed by the enzyme pyruvate kinase (PK) (Lunt and Vander Heiden, 2011). Under aerobic conditions pyruvate is transported into the mitochondria and converted to acetyl-CoA for OXPHOS. Under anaerobic conditions, lactate dehydrogenase (LDH) reduces pyruvate to lactate, which is then shunted into the extracellular space via the monocarboxylate transporter and then transported to the liver for gluconeogenesis (Salway, 2012).

In the presence of oxygen, mitochondrial acetyl-CoA generated via FAO or glycolysis enters the tricarboxylic acid (TCA) cycle where the acetyl group is transferred to oxaloacetate to form citrate. Through a series of well described reactions (Figure 1), citrate is converted first into its structural isomer, isocitrate and then α-ketoglutarate; reactions that lead to the production of NADH, H^+^ and CO_2_. Further decarboxylation of α-ketoglutarate liberates additional NADH and H^+^ and a high energy thioester, succinyl-CoA. Succinyl-CoA undergoes phosphorylation to form succinate and then further oxidation and hydration steps to reform oxaloacetate and additional NADH and H^+^. The NADH and H^+^ produced via the TCA cycle are then used to drive the mitochondrial electron transport chain (ETC) for the generation of ATP (Lunt and Vander Heiden, 2011). Clearly skeletal muscle metabolism is strictly regulated by substrate availability, presence of oxygen and energy demand, which in turn also regulate muscle protein metabolism and cell size.

## Metabolic disturbances leading to alterations in skeletal muscle mass

The preservation of skeletal muscle function is crucial for maintaining an independent lifestyle and the capacity to perform the activities of daily living. Generally considered to be the result of a balance between protein synthesis and degradation, skeletal muscle mass is carefully regulated through the actions of numerous complementary and (sometimes) interacting pathways. Any disruption to this careful balance of protein synthesis and degradation can have serious consequences. The role of metabolism in the progression of muscle wasting and weakness in individual disorders has previously been described in detail for diabetes, obesity (Akhmedov and Berdeaux, 2013), Pompe's disease (Raben et al., 2012), McArdles disease (Angelini and Semplicini, 2010) and several mitochondrial disorders (Russell et al., 2013). As such, our aim is to describe some of the more recent discoveries linking specific metabolic signaling pathways to muscle wasting and weakness, with a specific focus on the central role of mechanistic target of rapamycin (mTOR).

### The mTOR complex 1 signaling pathway regulates diurnal variations in protein synthesis

Protein turnover in skeletal muscle is highly responsive to changes in substrate availability (Rennie et al., 1982). It is generally accepted that acute changes in substrate availability, amino acids (AAs) in particular, modulate protein synthesis by altering mRNA translation. Many laboratories have shown that the signaling pathway involving mTOR complex I (mTORC1) plays a crucial role in the control of initiation and elongation of mRNA translation (Bodine et al., 2001; Bolster et al., 2003; Dreyer et al., 2006). The mTORC1 signaling pathway integrates a wide variety of extra- and intracellular signals, including insulin (and its related growth factors), nutrient (glucose and amino acids) availability, and cellular energy status to regulate protein synthesis, autophagy, cell growth and metabolism (Laplante and Sabatini, 2012). The activity of mTORC1 determines the activity of downstream effectors such as the 70-kDa S6 protein kinase (S6K1) and the eukaryotic initiation factor 4E-binding protein (4E-BP1) (Kimball et al., 2002). Both play key regulatory roles in modulating translation initiation, and control the binding of mRNA to the 40S ribosomal subunit (Kimball et al., 2002).

mTORC1 activity is controlled amongst others, by its upstream regulator, the tuberous sclerosis complex (TSC1-TSC2, Dodd and Tee, 2012). Activation of this complex stimulates the GTPase function of Rheb, a small GTPase that acts as a proximal key activator of mTORC1, which leads to a reduction in Rheb-induced mTORC1 activation. In contrast, inactivation of the TSC1-TSC2 complex results in the accumulation of GTP-bound Rheb and thus activation of mTORC1 (Dodd and Tee, 2012). Clearly, the activity of the TSC1-TSC2 complex and Rheb-mTORC1 interaction are critical for the correct operation of the mTORC1 pathway in response to changes in homeostasis.

Given that protein synthesis requires a plentiful supply of amino acids and energy (ATP), it is not surprising that mTORC1 signaling is under strict regulation. Increased availability of AAs strongly stimulates muscle protein synthesis (Rennie et al., 1982; Volpi et al., 1998, 1999; Paddon-Jones et al., 2004, 2006). Besides serving as a substrate for polypeptide biosynthesis, the essential AAs (EAAs), but not the non-essential AAs (NEAAs), have been shown to directly activate regulatory proteins in mRNA translation, thereby increasing muscle protein synthesis. Noteworthy, the latter event does not require increased NEAA availability (Volpi et al., 2003). The branched-chain AA, leucine, is of particular interest since it has the unique ability to directly increase signaling through mTORC1 and its downstream targets 4E-BP1 and S6K1 and ribosomal S6. Therefore, leucine represents the main anabolic signal responsible for the post-prandial increase in muscle protein synthesis (Smith et al., 1992; Norton and Layman, 2006).

Dickinson et al. (2011) have provided clear evidence that, in humans, rapamycin injection prior to EAA intake prevents the expected increase in protein synthesis and attenuates the increase in mTORC1-signaling, supporting a fundamental role for mTORC1 activation as a key-regulator of protein synthesis in response to increased AA availability. A detailed discussion about how cells sense AAs and how these signals are communicated to mTORC1 is beyond the scope of this review as we aim to focus in more detail how changes in glucose metabolism alter the activity of this particular pathway, instead the reader is directed to a number of recent excellent reviews (Dodd and Tee, 2012; Laplante and Sabatini, 2012).

One important example of the importance of mTOR in the metabolic regulation of muscle mass can be observed during the process of age-related muscle wasting and weakness (sarcopenia). While the effect of ageing and sarcopenia on mTOR associated signaling in skeletal muscle in the fasted state has been investigated in detail its role remains unclear. Some reports in humans suggest that mTOR and S6K1 protein expression (Cuthbertson et al., 2005) or phosphorylation status (Li et al., 2012) are reduced in muscles from elderly individuals, whereas others report no difference in the fasted state (Guillet et al., 2004; Drummond et al., 2008a). Importantly however, following the administration of EAA (with or without carbohydrates), elderly humans have a blunted increase in mTOR (Cuthbertson et al., 2005), and S6K1 (Guillet et al., 2004; Cuthbertson et al., 2005) phosphorylation compared with young controls, resulting in an attenuated (Cuthbertson et al., 2005) or delayed (Drummond et al., 2008a) anabolic response. These studies indicate that the skeletal muscle response to alterations in glucose and AA availability is compromised in the elderly, and could be a result of a defect in either the ability of the muscle to respond, or detect, a change in energy availability.

### AMP activated protein kinase as a negative regulator of skeletal muscle mass

The most well-studied energy sensor in skeletal muscle is the 5′-AMP-activated protein kinase (AMPK) (Steinberg and Kemp, 2009). AMPK is activated by an elevation in the AMP/ATP ratio leading to inhibition of energy consuming anabolic processes such as protein synthesis and stimulation of catabolic energy producing processes such as glycolysis, FAO and protein degradation (Steinberg et al., 2010). AMPK is thought to regulate mTORC1 signaling either via 1) phosphorylation of TSC2, leading to increased GTPase activity of Rheb; 2) direct phosphorylation of mTOR at Thr2446 preventing stimulatory phosphorylation on Ser2448; or 3) the phosphorylation and dissociation of the critical mTORC1 protein, Raptor (Steinberg and Kemp, 2009, Figure 2).

**Figure 2:**
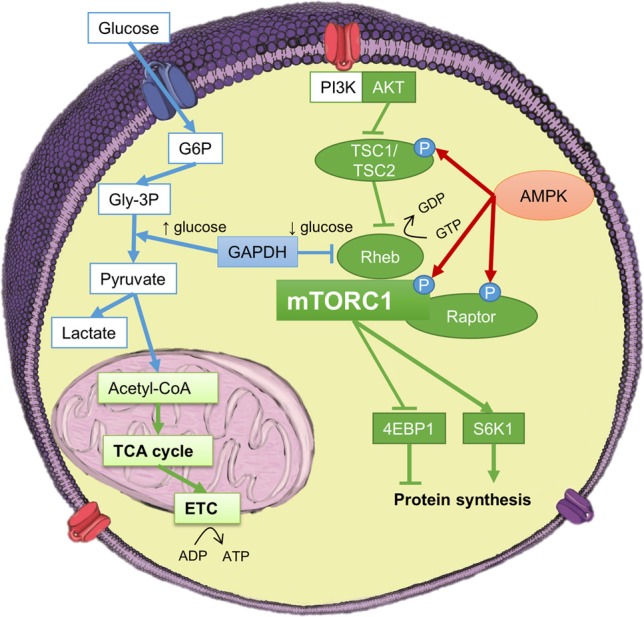
**Considered one of the master regulators of protein synthesis, mammalian target of rapamycin (mTOR) is perfectly positioned to receive feedback regarding the cellular energy status**. During levels of high glycolytic flux, the ADP/ATP ratio is low, and so AMPK activity is reduced, furthermore, GAPDH is prevented from interacting with the small GTPaseRheb. Together, these pathways lead to elevated mTOR complex I (mTORCI) signaling, and protein synthesis.

Pharmacological activation of AMPK using 5-Aminoimidazole-4-carboxamide ribonucleotide (AICAR) suppresses phosphorylation of mTOR and 4E-BP1 and induces atrophy in C2C12 myotubes *in vitro* (Zhao et al., 2010). On the other hand, knock-down of AMPKα1/2 subunits has been shown to increase myotube diameter, associated with a marked increase in S6K1 and protein synthesis rate (Lantier et al., 2010), an effect that was found to be ablated following treatment with rapamycin. In addition, skeletalmuscle-specific deficient AMPKα1/2 KO mice have increased muscle mass with bigger myofibers and S6K1 signaling (Lantier et al., 2010). AMPK activity is rapidly suppressed when muscles are exposed to increasing concentrations of either leucine or glucose that stimulate increases in muscle protein synthesis and signaling through mTORC1 (Saha et al., 2010). Conversely, activation of AMPK by AICAR reduced leucine- and glucose-stimulated increases in protein synthesis and mTOR phosphorylation (Saha et al., 2010). Clearly, AMPK can modulate mTORC1 signaling which is one of the mechanisms by which protein synthesis can be reduced during cellular stress.

Based on the described relationship between AMPK activity and signaling through mTOR, one would expect that reduced protein synthesis in metabolic diseases are associated with increased levels of AMPK activity. However, the role of AMPK in altered protein metabolism in sarcopenia, obesity and diabetes is unclear. Some reports demonstrate reductions in AMPK signaling in skeletal muscle samples collected from elderly humans (Li et al., 2012), whereas others report no change in the fasted state and increased AMPK phosphorylation following amino acid ingestion (Drummond et al., 2008b). In muscle samples from obese and type 2 diabetes patients, AMPK expression and activation are not significantly different from controls (Hojlund et al., 2004; Steinberg et al., 2004), suggesting that changes in AMPK signaling may not be the primary defect preceding metabolic changes associated with these conditions (Steinberg and Kemp, 2009).

### Glycolytic fluxin skeletal muscle can directly regulate mTORC1 activity

AMPK mediated signaling is not the only way cellular stress or a change in homeostasis signals to mTORC1 to regulate protein synthesis. Recently, it has been demonstrated that glycolysis is linked to the mTORC1 pathway via the direct binding of glyceraldehyde-3-phosphate dehydrogenase (GAPDH) to Rheb in HEK293 and mouse embryo fibroblasts (Lee et al., 2009). The GAPDH-mediated reaction in glycolysis is substrate limited, therefore, GAPDH is well suited to monitor the glycolytic flux. The glycolytic flux regulates the interaction between GAPDH and Rheb, and this interaction inhibits mTORC1 signaling by preventing Rheb from binding to mTOR (Dodson et al., 2013). GAPDH regulates the binding of Rheb to mTOR in a manner that is dependent upon glycolytic intermediates and is independent of the nucleotide-charged status of Rheb. High glycolytic flux suppresses the interaction between GAPDH and Rheb and thus allows Rheb to activate mTORC1, whereas low glycolytic flux enhances the binding of GAPDH and Rheb, ultimately suppressing mTORC1 signaling (Lee et al., 2009, Figure 2). Thus, the GAPDH-Rheb axis may be responsible for more close cross talk between the glycolytic and the mTORC1 pathways, whereas the AMPK-dependent pathways may be responsive to other conditions that alter the AMP/ATP ratio (Figure 2).

The idea that the rate of glycolysis controls more than just carbohydrate metabolism in muscle *in vivo* is supported by a recent study by Luo et al. (2013). These authors demonstrated that during the development and progression of colorectal cancer, expression of the secreted autophagy-inducing stress protein HMGB1 increased in the muscle of tumor bearing mice. HMGB1administration resulted in a reduction in the protein expression of pyruvate kinase muscle (PKM) isoform 1 leading to a reduction in PKM activity, which was associated with a reduced phosphorylation status of mTOR, increased autophagy, and increased utilization of AAs, glutamine in particular, to produce intermediates for the TCA cycle (Luo et al., 2013). These results are in line with previous observations from Saha et al. (2010) showing that increased glucose availability (25 vs. 5.5 mM) increased mTOR related signaling, independent of changes in ATP/AMP/ADP and creatine phosphate (Saha et al., 2010), but associated with increased lactate-to-pyruvate ratio. These data suggest 1) a higher flux through glycolysis; and 2) decreased NAD^+^-to-NADH ratio. These changes may suppress the interaction between GAPDH and Rheb and thus allow Rheb to activate mTORC1, and/or reduce the abundance of the NAD^+^-dependent histone/protein deacetylase SIRT1, ultimately reducing the activity of AMPK (Ruderman et al., 2010). The potential role of PKM and SIRT1in skeletal muscle regeneration will be discussed in more detail below.

### A novel role for non-essential amino acids in the regulation of protein metabolism and oxidative stress in skeletal muscle

Although NEAAs are generally believed not to be important for the regulation of protein synthesis under normal conditions, studies have indicated that some of these AAs can manipulate muscle protein metabolism during conditions of (chronic low-grade) inflammation or oxidative stress; e.g., during ageing (Wheeler et al., 1999; Roth et al., 2003). AAs such as glutamine and glycine are thought to modulate the production of inflammatory cytokines; thereby reducing the negative impact of these cytokines on protein metabolism.

Originally proposed to serve solely as a metabolic fuel or protein precursor for rapidly dividing cells, glutamine has been found to directly (or indirectly) regulate the expression of many genes related to metabolism, signal transduction, cell defense and repair, and to inhibit the activation of intracellular signaling pathways associated with cellular stress, such as the p38 MAP kinase and ERK pathways (for review see Curi et al., 2005). Examples of specific glutamine target molecules that help protect cells from inflammation and oxidative stress, include the increased expression of heat shock protein 72 and glutathione (Wischmeyer, 2006). Although the mechanism of action of glutamine has been studied in detail, the signaling mechanisms by which glycine can prevent or reduce cellular oxidative stress, and regulate protein synthesis/breakdown, metabolism, and the development of skeletal muscle, are not well understood.

Glycine is a simple NEAA consisting of a single carbon molecule attached to an amino and a carboxyl group. Glycine is often considered biologically neutral and sometimes used as an is onitrogenous control. However, evidence is emerging that glycine administration activates glycine-gated chloride channels in inflammatory cells, thereby effectively reducing [Ca^2+^]_i_, cytokine production, and whole-body (systemic) inflammation in several models (Zhong et al., 2003; Roth, 2007). Since increased inflammation plays a key role in the loss of skeletal muscle and adipose tissue with cancer cachexia, we recently tested the hypothesis that increasing glycine availability could represent a simple, safe and promising treatment to reduce wasting (Ham et al., 2013). We found that glycine treatment prevented ~50% of the cancer-induced loss in muscle mass and helped maintain muscle strength in tumor bearing mice. In addition, glycine reduced skeletal muscle IL-6 and F4/80 mRNA (a marker of macrophages) expression, and tended to reduce the oxidized glutathione/total glutathione ratio indicative of a reduction in oxidative stress. Finally, glycine treatment partially prevented the tumor-induced reduction in eIF-3f protein, a key protein in the regulation of mTORC1 binding to S6K1 (Lagirand-Cantaloube et al., 2008), normally seen in cachetic mice. These data suggest that during wasting conditions, the NEAA glycine can modulate anabolic signaling through mTORC1 (Ham et al., 2013). Clearly, glycine affects metabolism in multiple ways, but the exact cellular mechanisms of its action are not completely understood.

## Linking skeletal muscle metabolism to satellite cell biology and regeneration

In addition to the important role of metabolism in the regulation of protein balance and skeletal muscle mass, a developing body of literature has identified metabolism as playing an important role in the regulation of cell-fate during the specification and subsequent differentiation of stem-cells, a process that has been termed “metabolic reprogramming” (Lunt and Vander Heiden, 2011; Ryall, 2013).

### Skeletal muscle stem cells—the satellite cell

Skeletal muscle is capable of remarkable regeneration in response to injury or trauma, a property conferred on skeletal muscle by the presence of a resident population of stem cells, the satellite cell (SC, Brack and Rando, 2012; Relaix and Zammit, 2012; Yin et al., 2013). First identified by Alexander Mauro in 1961, the SC sits in a unique anatomical location between the sarcolemma of the muscle fiber and the basement membrane that envelops the fiber (Mauro, 1961). The physical space surrounding the SC is termed the “SC niche” (Bentzinger et al., 2013). Interestingly, SCs have been found to co-localize with blood vessels (Christov et al., 2007; Ryall, 2013), placing them in an optimal position to respond to intrinsic signals from both the skeletal muscle fiber itself and changes in the systemic environment.

In healthy adult skeletal muscle, the majority of SCs exist in a quiescent state, outside of the cell cycle, in a state termed G_0_. In response to injury or trauma, the SC leaves the quiescent state and enters the cell cycle at G_1_ (activation). The SC then becomes specified to the myogenic lineage (specification/commitment) and progresses through the cell-cycle (proliferation). After several rounds of proliferation, SCs exit the cell cycle and undergo differentiation and fusion to form an immature myotube. Finally, these myotubes mature and grow to form mature myofibers. In this manner, SCs can efficiently repair damaged muscle fibers. Importantly, a small population of SCs exit the cell cycle early and return to the G_0_ quiescent state, thus ensuring that the SC pool is replenished. Each of these steps is regulated through the coordinated expression of a family of transcription factors—the myogenic regulatory factors (MRFs); MyoD, Myf5, Myogenin, and MRF4 (MRFs, Brack and Rando, 2012; Bentzinger et al., 2013; Yin et al., 2013).

Although many other cell types such as PW1^+^ (Paternally expressed 3 protein)/Pax7^−^ interstitial cells (PIC), mesoangioblasts and mesenchymal stem cells have been proposed to contribute to myofiber regeneration (Dellavalle et al., 2007; Pannerec et al., 2013), work by Sambasivan and colleagues demonstrated that ablation of SCs led to failure of skeletal muscle regeneration (Sambasivan et al., 2011). These results suggest that while these “secondary” cell types contribute to regeneration they cannot replace the role of SCs. Thus, the remainder of this discussion will focus on SCs, as defined by the presence of the Pax7 transcription factor.

Advances in cell isolation techniques combined with the use of large scale gene arrays has provided a global view of quiescent SCs and insight into the regulation of SC quiescence and subsequent activation. Fukada et al. (2007) studied gene expression of quiescent and proliferating SCs, by combining fluorescence activated cell sorting (FACS) to isolate a pure subpopulation of SCs, followed by microarray analyses on either freshly isolated SCs (quiescent) or *ex vivo* activated and proliferating SCs. SCs were FACS isolated from a mononuclear suspension using fluorescently labeled antibodies for SM/C-2.6 (target antigen currently unknown) and CD45, with SM/C-2.6^+^ and CD45^−^ cells defined as the SC population. Utilizing microarray technology, 507 genes were identified with greater than five-fold differential expression in the quiescent vs. the proliferating SC populations. As expected, genes involved in the negative regulation of cell cycle progression were enriched in quiescent SCs. Interestingly, genes encoding regulators of cell-cell adhesion molecules, resistance to oxidative stress, and lipid transporter activity were also enriched in quiescent SCs (Fukada et al., 2007). It has been proposed that signaling through cell-cell adhesion molecules maintains SCs in an undifferentiated quiescent state, while resistance to oxidative stress is essential for all stem cell populations, so as to prevent free radical induced damage to the DNA. However, the importance of lipid transport and FAO in quiescent SCs has yet to be investigated.

In support for a role of FAO in the regulation of a stem-cell population, Ito and colleagues have shown that FAO may play a role in regulating hematopoietic stem-cell (HPSC) fate decisions (Ito et al., 2012). In this study the authors focussed on the role of peroxisome proliferator-activated receptor δ (PPARδ), which has been implicated in nutrient sensing and transcriptional regulation of genes involved in FA transport and FAO during stem-cell self-renewal (Takahashi et al., 2007). The inhibition of PPARδ or FAO in the context of HPSCs led to a decrease in self-renewal, and a decrease in the ratio of asymmetric to symmetric division in these cells. In contrast, treatment of HPSCs with a PPARδ agonist improved the maintenance of the HPSC population and increased the proportion of asymmetric divisions (Ito et al., 2012). This exciting study provided evidence that the PPARδ-FAO pathway may play an important role in the control of stem cell fate and, in particular, the control of asymmetric division of HPSCs.

### Metabolic reprogramming—linking metabolism to transcription and the regulation of stem cell fate

The first evidence linking a change in cellular metabolism to a change in cell state was provided by Otto Warburg in 1956, who found that tumor cells preferentially utilized the glycolytic pathway even in the presence of oxygen (Warburg, 1956). This process was referred to as aerobic glycolysis, and later the “Warburg effect.” Since this seminal finding a significant body of work has focussed on the altered metabolism that occurs in tumor cells, and it has recently been proposed as a core hallmark of cancer (Ward and Thompson, 2012). Interestingly, a process of metabolic transformation has been identified in stem-cell populations during changes in cell-fate, with an explosion of interest in this area over the last 2–3 years. Through advances in developmental, cancer and stem-cell biology, it has become apparent that changes in cellular metabolism play a large role in the regulation of stem cell function—a process termed “metabolic reprogramming.” Studies in embryonic stem cells (ESCs), and induced pluripotent stem cells (iPSCs) revealed that in these highly proliferative populations there is an increased reliance upon glycolysis and a reduced level of OXPHOS, compared with cells undergoing differentiation (Folmes et al., 2011; Zhang et al., 2011, 2012). The increased reliance on glycolysis has been attributed to the requirement of these cells to have access to a large supply of carbon and nitrogen for the generation of new biomass in these proliferating cells (Lunt and Vander Heiden, 2011, Figure 3). In contrast, studies in adult stem cell populations (that exist in a quiescent state, such as human T cells and resting B cells) have indicated that these cell populations rely upon FAO and OXPHOS, and upregulate markers of glycolysis upon a shift toward active proliferation (Wang et al., 2011; Le et al., 2012).

**Figure 3:**
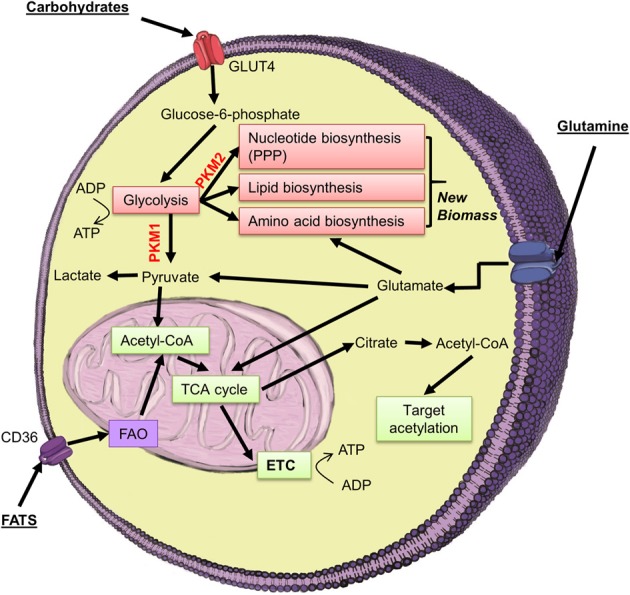
**Highly proliferative cell populations, such as some tumors, ESCs, iPSCs, and SCs require a ready supply of carbon and nitrogen for the generation of new biomass (nucleotides, proteins, phospholipids)**. To achieve this, many highly proliferative cell populations switch to a predominantly glycolytic based metabolism, but upregulate the PKM2 splice isoform of pyruvate kinase. In this manner, proliferating cells can build up sufficient glycolytic intermediates for the biomass necessary for cell division.

Clearly, the energetic demands and need for new biomass will differ for SCs during periods of quiescence, proliferation and differentiation. Thus, SCs must reprogram their metabolic profile to match these altered conditions. Evidence of a link between metabolism, SC identity and transplant efficiency has been provided by a recent study focussed on caloric restriction (CR, Cerletti et al., 2012). Mice were given a diet consisting of 60% of the caloric intake of standard *ad lib* fed mice for 12 weeks. At the completion of this dietary intervention there was an increase in total SC number, increased SC mitochondrial abundance and OXPHOS activity, and an increased proliferative capacity of SCs. Furthermore, the increase in OXPHOS activity observed in SCs isolated from CR mice was associated with a two-fold increase in the transplant efficiency of these cells (Cerletti et al., 2012). Interestingly, when control SCs were transplanted into a CR host animal there was a similar improvement in transplant efficiency; indicating that both intrinsic SC factors and the host environment may influence the overall efficacy of SC transplant therapies (Cerletti et al., 2012).

The “golden age of biochemistry” in the first half of the 20th century helped define the majority of the metabolic pathways responsible for nutrient breakdown, however, it is only recently that a potential link between metabolism and cellular identity has been proposed (Deberardinis et al., 2008; Daley, 2012; Deberardinis and Thompson, 2012). As the metabolic state of a cell reflects the integrated response to both intracellular energy demands and the extracellular environment, alterations to either can lead to changes in metabolite balances (NAD^+^/NADH, ADP/ATP, GDP/GTP), cellular pH, oxygen availability, small molecules (acetyl-CoA, methionine), voltage gradients and many more. All of these metabolically regulated changes can lead to differential regulation of transcription, and changes in cell identity (Lu and Thompson, 2012).

#### Acetyl-CoA and histone acetylation

Chromatin structure and organization is carefully regulated through a series of dynamic post-translational modifications, including (but not limited to) acetylation, methylation, phosphorylation, and ubiquitination. These modifications can alter the accessibility of the DNA to transcription initiating factors. One common histone modification, acetylation, has been found to be regulated in a metabolic dependant manner (Wellen et al., 2009; Lu and Thompson, 2012). Histone acetylation occurs via the actions of a histone acetyltransferase (HAT) in a reaction that transfers the acetyl group from acetyl-CoA to a specific residue (typically lysine) on the histone tail. In a study by Wellen et al. (2009) glucose was found to be the primary source of acetyl-CoA used in histone acetylation via conversion of mitochondrial derived acetate, induced by the enzyme ATP-citrate lyase (ACL). In this study, the authors used siRNA to demonstrate that ACL is essential for histone, but not protein acetylation. These results suggest that the acetyl pool used for protein acetylation may not be the same as that used for histone acetylation (Rathmell and Newgard, 2009). Importantly, a switch in substrate availability/utilization could lead to a rapid alteration in acetyl-CoA availability and histone acetylation status. Whether such a switch exists in SCs, and what role it may have, remains an intriguing possibility.

#### NAD^+^ and the sirtuin family of histone/protein deacetylases

The class III family of histone/protein deacetylases, the sirtuin family, consists of seven members, all of which contain a conserved core catalytic domain and differ in their C- and N-terminal domains (Ryall, 2012). Unlike class I and II histone deacetylases (HDACs), sirtuins require NAD^+^ for their deacetylase activity. One of the best described sirtuins, SIRT1 acts as a catalyst to transfer the acetyl group of the protein target to NAD^+^ to produce nicotinamide (NAM), 2′-O-acetyl-ADP ribose, and the deacetylated target protein. Due to the reliance upon NAD^+^, SIRT1 can be considered as an “energy sensor” that is activated in response to an increase in NAD^+^ availability. At the level of whole skeletal muscle SIRT1 has been found to target a range of histone and protein targets, including histones H3K9 and H4K16, and the transcription factors PGC1α, MyoD, and FoxO1/3a (Fulco et al., 2003, 2008; Vaquero et al., 2006).

In 2003 Fulco and colleagues identified SIRT1 as an important regulator of skeletal muscle gene expression (Fulco et al., 2003). In this study the authors demonstrated that increased SIRT1 activity lead to inhibition of C2C12 differentiation, and reduced the expression of Myogenin (*Myog*) a master regulator of differentiation. In a follow up study, these authors went on to demonstrate that during periods of reduced nutrient availability, C2C12 differentiation was inhibited in both a SIRT1 and NAD^+^ dependent manner. Interestingly, the NAD^+^ salvage enzyme nicotinamide phosphoribosyltransferase (Nampt, responsible for the conversion of nicotinamide back to NAD^+^) was found to mediate the effects of nutrient deprivation on myogenic differentiation (Fulco et al., 2008). However, the role of SIRT1 (and indeed NAD^+^) has yet to be investigated in SCs, particularly during important cell fate decisions such as myogenic commitment and the process of differentiation.

While SIRT1 is the best described of the sirtuin family (in the context of skeletal muscle), there exists six other mammalian sirtuins all of which have been found to regulate metabolism in many tissues (Chang and Guarente, 2013). Currently, very little is known about the role (if any) of SIRT2-6 in SCs, however a number of recent studies have begun to identify a role for some of these deacetylases in skeletal muscle. SIRT3 is localized to the mitochondria and has been found to promote the activity of a number of important mitochondrial enzymes, including pyruvate dehydrogenase, in a deacetylation dependent manner (Fernandez-Marcos et al., 2012; Jing et al., 2013). The SIRT4 isoform (also localized to the mitochondria) can regulate lipid metabolism via deacetylation of the malonyl CoA decarboxylase (MCD) enzyme leading to its inhibition and subsequent lipogenesis (Laurent et al., 2013). Finally, SIRT6 has been found to (indirectly) negatively regulate AKT phosphorylation, and subsequent hypoglycaemia via increased transport of glucose into skeletal muscle (Xiao et al., 2010).

#### Serine/glycine metabolism and histone methylation

Similarly to the requirement for acetyl-CoA for histone acetylation, histone methylation requires S-adenosyl (SAM) methionine as a methyl-donor. Shyh-Chang and colleagues have recently demonstrated a requirement for the amino acid threonine in histone H3K4 trimethylation (H3K4me3) in pluripotent mouse embryonic stem cells (mESC, Shyh-Chang et al., 2013). Interestingly, these authors determined that threonine was a requirement for H3K4me3 and H3K4me2, but not H3K4me1, H3K9me3, H3K27me3 or H3K26me3, suggesting that threonine levels regulate the methylation status of specific lysine residues. However, similarly to acetyl-CoA metabolism, the role of threonine metabolism in SC quiescence, proliferation and differentiation has not been investigated.

Utilizing chromatin immunoprecipitation, followed by whole genome sequencing (ChIPseq), it is possible to obtain a global enrichmentprofile of specific histone modifications. Liu and colleagues used ChIPseq to analyse the global expression profile of histone H3K4me3 and H3K27me3 in FACS isolated quiescent and proliferating SCs (Liu et al., 2013), and found that while both quiescent and proliferating SCs exhibited similar H3K4me3 profiles, the global level of H3K27me3 was significantly enriched. As expected (due to its link to gene silencing) many genes that were down regulated in proliferating SCs exhibited a dramatic enrichment of H3K27me3 throughout the gene body. In a previous study by Juan and colleagues, the histone methyltransferase Ezh2 was found to be an essential regulator of SC identity and self-renewal. While not present in quiescent SCs, Ezh2 was rapidly upregulated in activated and proliferating SCs, leading to H3K27me3 and inhibiting the expression of transcription factors known to regulate non-myogenic lineages (Juan et al., 2011).

#### Differential splicing of pyruvate kinase, and histone phosphorylation

Differential splicing of PKM at exons 9 and 10 has been found to be an important regulator of the decision to shunt glycolytic intermediates for breakdown to acetyl-CoA (which will enter the mitochondria and the TCA cycle), or to instead enter the PPP to produce nucleotides, proteins and phospholipids for cell growth (Gupta et al., 2011; Macintyre and Rathmell, 2011). Inclusion of exon 9 produces PKM1, which catalyzes the dephosphorylation of phosphoenolpyruvate (PEP), and promotes the entry of pyruvate into the mitochondria for conversion to acetyl-CoA. In contrast, exon 10 inclusion produces the PKM2 splice isoform which has a reduced affinity for PEP, and leads to the build-up of glycolytic intermediates available for entry into the PPP (Gupta et al., 2011). Interestingly, highly proliferative stem-cells such as ESCs and tumor cells exhibit preferential transcription of PKM2—indicating that PKM may play an important role in the process of stem-cell metabolic reprogramming (Lv et al., 2011; Ye et al., 2012). Similarly, proliferating C2C12 cells exhibit preferential transcription of the PKM2 splice isoform, which has been proposed to be essential to allow the cells to generate sufficient intermediates for the generation of new macromolecules (Harada et al., 1995; David et al., 2010).

### A link between skeletal muscle fiber metabolism and satellite cell density

The space that surrounds the SC between the basal lamina and sarcolemma has been termed the “SC niche” (Lander et al., 2012). The majority of adult stem-cells have been found to localize to a specialized niche, and a number of exciting studies have proposed that SC function can be regulated via changes to the niche environment (Gilbert et al., 2010; Chakkalakal et al., 2012). It is interesting to postulate that the metabolic milieu of the SC niche may be different from that of the muscle fiber and/or the extracellular space. Thus muscle damage would be expected to destroy the niche and expose the SC to an altered metabolic environment, leading to rapid changes in both nutrient uptake and intracellular metabolism.

In addition to the local niche milieu (open to influence via changes in the systemic environment), SC numbers can be influenced by the fiber they are attached to, with an increased number of SCs associated with fibers that are predominantly oxidative (slow, type I fibers), compared with fibers that rely primarily on glycolysis (fast, type II fibers) (Putman et al., 2001; Christov et al., 2007). However, whether this is due to direct signaling from the fiber to the SC population and what role the metabolic status of the fiber may play in SC biology, has yet to be investigated.

Both physiological and pathological changes in metabolism can influence stem-cell number and function. Interestingly, interventions that promote a shift in skeletal muscle metabolism from glycolysis to OXPHOS, such as chronic low-frequency stimulation (LFS) of the peroneal nerve (a model of endurance exercise training), have been observed to lead to an increase in SC number (Putman et al., 1999, 2001). While LFS is a well characterized model in regards to effects on whole muscle and single fiber metabolism, very little is known regarding the effects on SC metabolism.

## Conclusions

While a wealth of information exists on the role of metabolism in health and disease, it is only more recently that we are beginning to appreciate the close link between metabolism and skeletal muscle wasting and regeneration. The studies presented in the current discussion have identified numerous ways in which metabolism can directly influence protein synthesis and transcription. It is in this manner that metabolic remodeling can play a large role in both physiologic and pathologic adaptations during a disruption in homeostasis. However, it is also clear that significant questions remain regarding the role of metabolism in skeletal muscle, particularly with reference to its role in regulating SC biology and skeletal muscle regeneration.

### Conflict of interest statement

The authors declare that the research was conducted in the absence of any commercial or financial relationships that could be construed as a potential conflict of interest.
